# Supplementary material for: Pupil-linked arousal signals track the temporal organization of events in memory
Source: Nat Commun. 2020 Aug 11;11:4007. doi: 10.1038/s41467-020-17851-9 (PMC7421896; doi:10.1038/s41467-020-17851-9)
Supplement: Supplementary file 1 — Supplementary Information [file 41467_2020_17851_MOESM1_ESM.pdf]

Supplementary Information

Pupil-linked arousal signals track the temporal organization of events in memory

David Clewett, Camille Gasser & Lila Davachi

Supplementary Methods

Linking the two memory-related pupil components to task-relevant features of the encoding task. A qualitative PCA decomposition of the pupil component loadings by condition revealed that loadings may vary according to the different stimulus types. To quantify these effects, we recomputed the PCA using a 1.5s window for the boundary trials and same-context trials, separately. Examining this short pupil-sampling window enabled us to target the effects of the tones on pupil dilation prior to the onset of the images. Average baseline-normed pupil dilation was computed for each of the tone types (boundary and same-context trials). These values were then averaged across participants to reduce the amount of input variables (130 variables; 65 participants with two conditions each). All pupil samples across a 1.5-s window covering the average time-course of tone-evoked pupil dilation (375 pupil samples in total; see **Supplementary Figure 2a**) served as dependent measures in the PCA. As before, an unrestricted PCA using the covariance matrix with Varimax rotation and Kaiser normalization was used on all components. Factor loadings with eigenvalues greater than 1 were analyzed in subsequent analyses (Kaiser criterion; [1]).

The 1.5-s window pupil PCA revealed three components that accounted for significant variance in tone-evoked pupil dilations (**Supplementary Figure 2a**). Importantly, these three pupil components shared substantial overlap with three of the pupil components identified in the main 3-s PCA window analysis. For illustrative purposes, the pupil components from both PCAs are displayed in the same blue colors (for comparison of their overlap, see **Figure 5c,d** in main text and **Supplementary Figure 2)**. The temporal features of these components, including their latencies-to-peak and the amount of variance they accounted for, were as follows: (1) an intermediate component (1,500ms; 85.77% variance; royal blue); (2) a slowly-decreasing component (520ms; 11.16% variance; sky blue); and (3) an early-peaking component (800ms; 1.90% variance; turquoise). As before, we also performed separate follow-up PCAs on the 1.5s boundary trials and same-context trials to illustrate qualitative differences in their loadings over time.

As shown in **Supplementary Figure 2b**, two-tailed paired t-tests between the loadings on boundary and same-context trials from the 1.5s PCA revealed that boundaries significantly modulated loadings on the intermediate pupil component (royal blue; $t(64) = 9.45$, $p < .001$, d = 1.17, [CI: 0.92, 1.41]) and the early-peaking pupil component (turquoise; $t(64) = 3.51$, $p < .001$, d = 0.44, [CI: 0.21, 0.76]). When these data were analyzed separately for the two experiments, we found that boundaries significantly modulated loadings on the intermediate pupil component

(royal blue; t(29) = 6.02, p < .001, *d* = 1.10, [CI: 0.74, 1.50]) and the early-peaking pupil component (turquoise; t(29) = 3.57, p < .001, *d* = 0.65, [CI: 0.23, 0.84]) in Experiment 3. In Experiment 2, boundaries again significantly modulated loadings on the intermediate pupil component (t(34) = 7.23, p < .001, *d* = 1.22, [CI: 0.86, 1.54]), but only did so marginally significantly for loadings on the early-peaking pupil component (t(34) = 1.97, p = .057, *d* = 0.32, [CI: -0.014, 0.89]).

It is noteworthy that, unlike the 3-s PCA that accounted for the combine pupil responses to tones and images, the tone switches alone (1.5-s window) did not modulate loadings on the slowly-decreasing, 'anticipatory' component across studies (sky blue; t(64) = 0.90, p = 0.37, *d* = 0.11, [CI: -0.098, 0.26]), or in Experiment 2 (t(34) = 1.24, p = 0.22, *d* = 0.21, [CI: -0.11, 0.44]) or Experiment 3 (t(29) = -.19, p = 0.37, *d* = .035, [CI: -0.25, 0.21]), separately. This result suggests that pupil-linked anticipation following a tone switch is present to a similar degree for 'boundary' images and images from the same-context **(**for comparison, see **Figure 6d).** We interpret this result as being consistent with evidence suggesting that the context shift and the boundary items are both processed differently than same-context information, with anticipation and top-down processes being important for segmenting subsequent memories.

Trial-level fluctuations in phasic and tonic arousal levels as mechanisms of memory separation. The PCA uncovered evidence that different temporal characteristics of boundary-evoked pupil dilation were related to memory performance *across participants*. However, it is also possible that more discrete, trial-level changes in tonic (global) and/or phasic (transient, stimulus-evoked) arousal states contribute to event segmentation in memory. This idea is inspired by evidence that tonic arousal states also influence memory and decision processes [2]. To explore these possibilities, we examined whether trial-level changes in phasic (indexed by tone-evoked pupil dilation) and tonic (indexed by average pupil size during the pre-tone baseline period) arousal were associated with subsequent memory outcomes**.** To test this, we conducted separate hierarchical linear modeling (HLM) analyses using the lmer function in the lme4 library (see **Supplementary Figure 3a,b**). Parameters were estimated with the maximum likelihood method in R (R Core Team, 2012). This approach is exceptionally sensitive to linear modulation of memory outcomes by trial-level variations in pupil diameter.

In the phasic arousal analysis, we computed the average boundary-related pupil dilation response by subtracting the average pupil size within a 500-ms window prior to tone onset from the average pupil size 1-2s post-tone onset. In the tonic arousal analysis, we examined the relationship between average pupil diameter during this 500ms pre-tone baseline period and

subsequent memory. Model fits included random intercepts and slopes for the pupil predictor by participant, enabling us to capture individual differences in pupil-memory associations. These analyses were performed on boundary trials only.

The results revealed no significant relationships between pupil diameter prior to the boundary tones and subsequent source or temporal memory in any of the experiments (all p's > .05; **Supplementary Figure 3a,b**). These findings suggest that trial-level pupil-averaging might obscure more nuanced arousal/pupil mechanisms detected by PCA, and lend additional credence to the importance of using PCA decomposition approaches to dissociate sub-components of the pupil dilation response.

The Relationships between the magnitude of a context shift at boundaries, pupil dilation, and subsequent memory. Another possibility we explored was whether the magnitude, or degree of salience, of the tone switch predicts the likelihood of memory segmentation later on. For this approach, we leveraged the varying magnitude of changes in tone pitch across boundaries (e.g., 500Hz pre-boundary tone to an 800Hz boundary tone) to see whether the 'strength', or salience, of an event boundary has an effect on later temporal and source memory **(Supplementary Figure 4a)**. This enabled us to leverage the sensitivity in trial and participant-level variability in tone pitch transitions across boundaries, which were randomized across individuals and lists. For example, the tone could switch from 500Hz to 800Hz at boundaries, reflecting a tone change of 300Hz. We computed these pitch-change predictors as absolute values, because we did not have strong predictions about whether an increase or decrease in pitch at boundaries would have different effects on memory (**Supplementary Figure 4a)**. Because there were 6 pitch options (ranging from 500Hz to 1000Hz), these values ranged from 100Hz to 500Hz.

As in the prior analysis, separate hierarchical linear modeling (HLM) analyses were carried out using the lmer function (see **Supplementary Figure 4a**) and parameters were estimated with the maximum likelihood method in R (R Core Team, 2012). Absolute values for tone pitch changes from pre-boundary to boundary tones were extracted for each boundary trial and mean-centered within each participant. These were modeled as level-1 predictors, and model fits included random intercepts and slopes for pitch change by participant. We also performed a separate analysis to see if pitch changes were correlated with arousal spikes at boundaries, indexed by average pupil dilation to the boundary tones (see **Figure 4a**).

The results revealed no significant association between tone-pitch changes at boundaries and pupil dilation at those boundaries. There was also no significant relationship

between pitch change at boundaries and memory outcomes for boundary items, suggesting that the degree of context change did not elicit different levels of arousal or modulate episodic memory **(**(all p's > .05; **Supplementary Figure 4b).** These findings suggest that a simple context shift, or boundary, is sufficient to drive memory separation irrespective of its magnitude. However, this does not exclude the possibility that the range of pitches (500Hz-1000Hz) in these experiments weren't variable enough to detect sensitivity in the pupil response to boundaries and/or behavior. Furthermore, at least on the temporal order memory test, performance was near floor. This raises the possibility that there was also limited variability in performance levels, which reduced our ability to detect meaningful effects of discrete pitch changes at boundaries.

The temporal stability of arousal serves as a mechanism of memory integration. The pupil dilation results from our PCA decomposition suggest that a spike in arousal at event boundaries can indeed promote event segmentation in later memory. In a more exploratory type of pupil analysis, we asked if prolonged fluctuations in arousal over more prolonged periods of time support temporal memory integration. Here we reasoned if arousal itself is an internal contextual state, it should also serve as a proxy for the stability of ongoing cognitive operations as experiences unfold. Through this lens, we predicted that greater stability in arousal over relatively long periods of time (e.g., 19+ seconds) would promote stronger temporal memory integration.

Here, we used a separate analysis approach that enabled us to leverage the rich time-course of arousal data on the order of tens of seconds on a trial-by-trial basis. Specifically, we performed linear mixed effects regression analyses to examine the relationship between trial-by-trial variability in pupil diameter between the to-be-tested item pairs and the two subsequent temporal memory outcomes (see **Supplementary Figure 5, top panel**). To quantify the stability of pupil-linked arousal fluctuations during sequence learning, we measured the standard deviation in pupil diameter between the onset of the first item from a to-be-tested memory pair and the offset of the second item from that pair (see **Supplementary Figure 5, top panel**). Across the two eye-tracking studies, the temporal distance between the to-be-tested item pairs were as follows: Experiment 2 = 19 seconds (4,750 pupil samples) and Experiment 3 = 30 seconds (7,500 pupil samples).

To query the relationship between this trial-level pupil variability measure and episodic memory, we again performed separate hierarchical linear modeling (HLM) analyses using the glmer function in the lme4 library. The standard deviation values of pupil sizes between the to-

be-tested image pairs were mean-centered within each participant and modeled as level-1 predictors. Model fits included random intercepts and slopes for pupil variability (i.e., standard deviation) by participant, enabling us to capture individual differences in pupil-memory associations. Condition (Boundary vs. Same-Context) was also entered as a fixed-effect predictor in each model. In separate linear mixed effects regressions, we examined the relationship between pupil diameter variability and temporal aspects of memory, namely temporal recency discriminations and distance ratings.

A two-tailed paired t-test revealed no main effect of event boundaries on pupil size variability ($p > .05$). This is perhaps unsurprising, given that the average tone-evoked pupil dilation lasted approximately 3 seconds (see **Figure 5a**). This window only covered ~16% of temporal interval between to-be-tested item pairs in Experiment 2 and only 10% of the time interval between item pairs in Experiment 3. Thus, the data were collapsed across conditions (boundary vs. same-context).

We first examined the relationship between pupil-linked arousal stability and an objective measure of temporal memory: temporal recency discrimination performance. Across both eye-tracking experiments (Experiments 2 and 3), we found that lower pupil size variability between a given pair of items was associated with more accurate recency discriminations of those items ($z = -2.09$, $p = .037$; **Supplementary Figure 5, bottom left**). When the experiments were analyzed separately, this relationship was significant in Experiment 2 ($z = -2.55$, $p = .011$), which had a shorter interval between to-be-tested item pairs. However, we did not observe this relationship in Experiment 3, which had a longer time interval between to-be-tested item pairs at encoding ($z = -0.23$, $p = .82$). Thus, at least at the shorter timescale examined in Experiment 2 (i.e., 19 seconds), it appears that pupil-linked arousal stability may support temporal order memory.

Next, we examined the relationship between arousal stability and a subjective measure of temporal memory: temporal distance ratings. Collapsing across both Experiments 2 and 3, we found that lower pupil size variability between a given item pair was associated with more compressed retrospective estimates of temporal distance between that same item pair ($t = 2.05$, $p = .040$; **Supplementary Figure 5, bottom right**). This effect was smaller and trended towards statistical significance in both Experiment 2 ($t = 1.48$, $p = .14$) and also in Experiment 3 ($t = 1.57$, $p = .12$) when the two experiments were analyzed separately.

Together these regression results support the idea that stable pupil-linked arousal states across time may provide and/or signal a form of internal context that supports binding of sequential information in memory. The results from the temporal order memory analysis suggest

that arousal stability may help to link successive information together in memory. However, this memory integration may only occur at relatively shorter timescales of encoding, as this relationship was not observed when item pairs were studied farther apart in the sequence (i.e., in Experiment 3). The results from the temporal distance ratings analysis suggest that pupil-related arousal stability may also facilitate the creation of tight-knit and temporally-compressed episodic memories. This pattern was consistent across both studies, suggesting that the objective interval between two to-be-associated items might not moderate the strength of this subjective memory effect.

Supplementary Figures

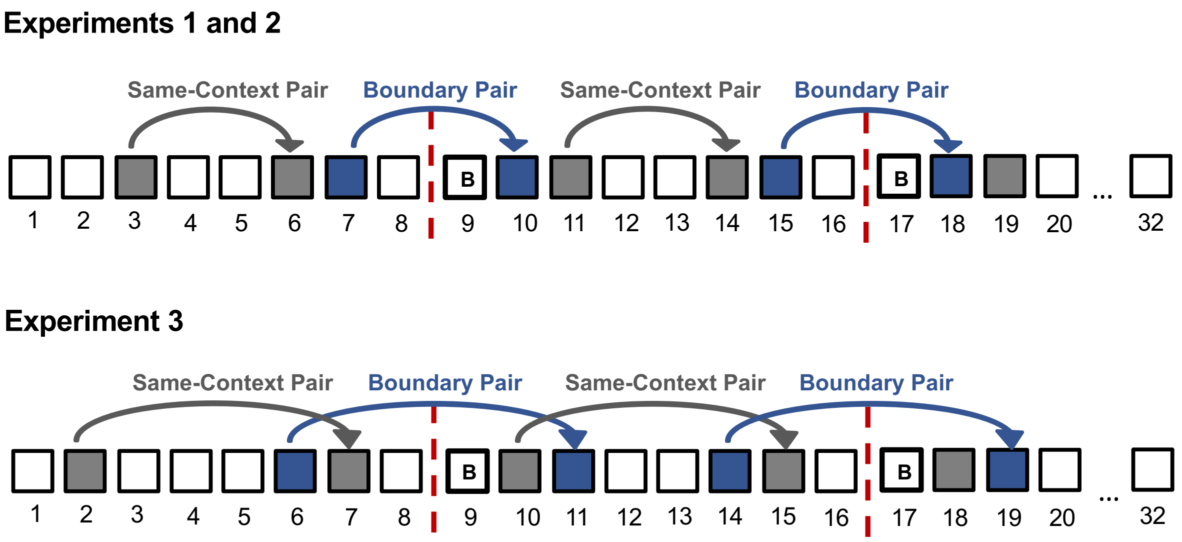

Supplementary Figure 1. Schematic depicting the item positions for which different aspects of memory were tested after each 32-item slideshow. Colored boxes and arrows represent the positions of item pairs that were later tested in the temporal order memory and temporal distance memory tests. In Experiments 1 and 2, all of the tested item pairs had been presented with two intervening items during encoding. In Experiment 3, all of the tested item pairs had been presented with four intervening items during encoding. Dashed lines represent the location of event boundaries, or tone switches, which always occurred after 8 successive items. White boxes represent the positions of the individual items that were later tested for source memory (i.e., the ear that the accompanying tone played in). Boxes with a “B” signify boundary items, or the object images that appeared just after an event boundary. The remaining white boxes represent same-context items, except for the first and last items in each list, which were analyzed separately.

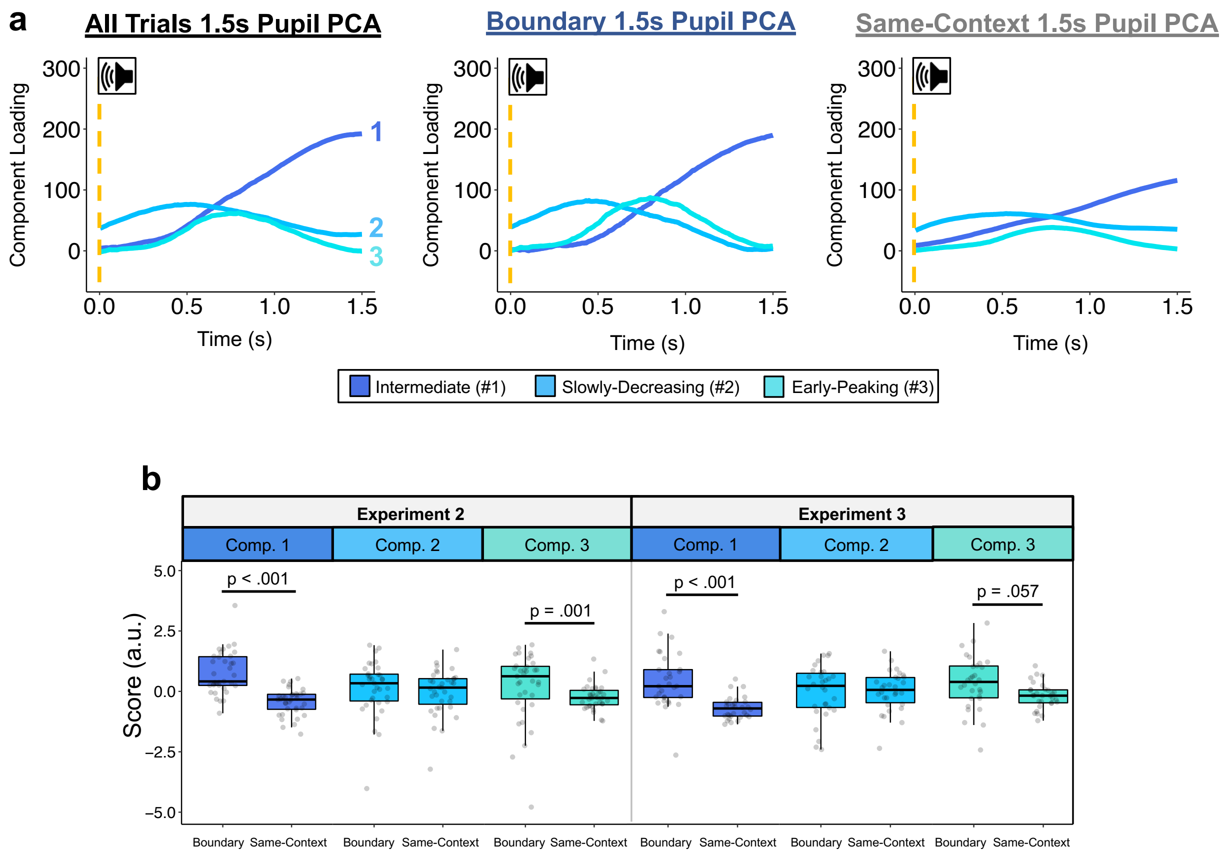

Supplementary Figure 2. Tone-specific effects on different temporal characteristics of pupil dilation. (a) Temporal features of tone-evoked pupil dilation identified by a temporal principal component analysis (PCA) focused on the 1.5s tone period. For illustrative purposes, separate PCAs were performed on the boundary and same-context trials to show qualitative differences in pupil loadings across time. Vertical dashed lines signify the onsets of the tones and their subsequent images. The 1.5s PCA revealed three significant features of pupil dilation that had distinct shapes over time and matched three of the components identified in the 3-s window PCA. Component factor loadings reflect “raw” values from the rotated solution, so are on the same scale as the original pupil inputs. (b) Condition-related differences on pupil component loadings. The boundary tones (blue colors) versus same-context tones (gray) differentially modulated loading scores for component #1 (intermediate) and component #3 (early-peaking), but not for component #2 (slowly-decreasing). All error bars represent standard error of the mean (SEM). Overlaid dots represent individual participants (Experiment 2: $n = 35$; Experiment 3: $n = 30$). Two-tailed paired pairwise t-tests were performed to test for significant differences between loadings for boundary trials compared to same-context trials. No adjustments were made for multiple comparisons. The pupil component loading results the first eye-tracking experiment were replicated in the third experiment, with the exception of component 3; this result was marginally significant in the third experiment. Source data are provided as a Source Data file.

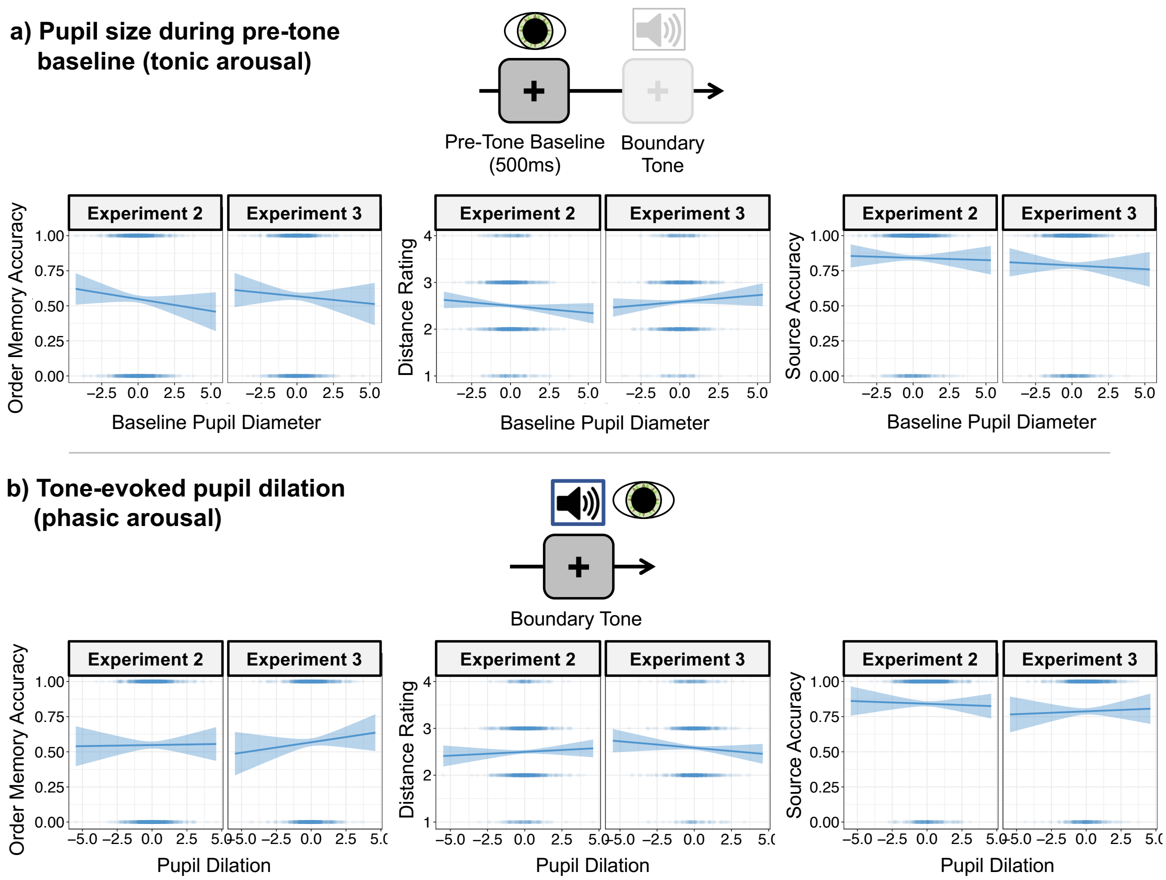

Supplementary Figure 3. The relationship between trial-level pupil measures of tonic and phasic arousal and subsequent memory broken down by eye-tracking experiment. (a) To assess global, or tonic, levels of arousal just prior to a boundary, pupil diameter values were averaged across the 500ms baseline period preceding a tone switch. Mixed effect linear modeling revealed no significant associations between baseline pupil diameter and any of the three memory outcomes. (b) To assess stimulus-evoked, or phasic, levels of arousal induced by boundaries, pupil diameter values were averaged across the window 1-2s after the onset of tone switch. These values were then baseline-normalized by subtracting the average pupil diameter in the 500ms prior to the onset of the tone switch. Mixed effect linear modeling revealed no significant associations between tone-evoked pupil dilation and any of the three memory outcomes. Shaded blue areas represent 95% confidence intervals. Overlaid dots represent individual participants (Experiment 2: $n = 35$; Experiment 3: $n = 30$). Source data are provided as a Source Data file.

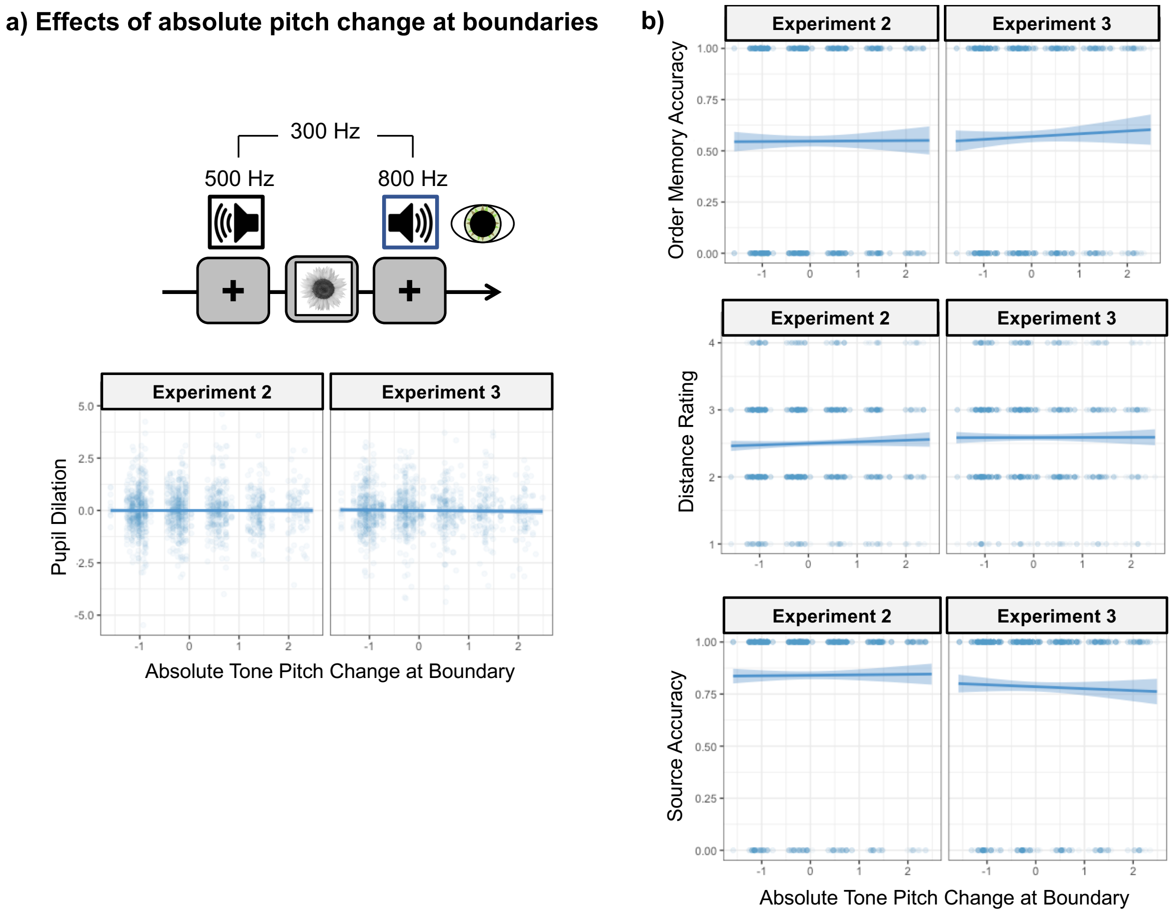

Supplementary Figure 4. Relationships between the magnitude of a tone-related context shift, subsequent memory, and tone-evoked pupil dilation. (a) The magnitude of a tone-related context shift was quantified by measuring the absolute change in the pitch (frequency) of the tones for the pre-boundary tone to the boundary tone. Mixed effect linear modeling revealed no significant association between the pitch change at boundaries and tone-evoked pupil dilation. (b) In addition, changes in tone-pitch at boundaries did not correlate with any of the three memory outcomes. Hz = Hertz. Shaded blue areas represent 95% confidence intervals. Overlaid dots represent individual participants (Experiment 2: $n = 35$; Experiment 3: $n = 30$). Source data are provided as a Source Data file.

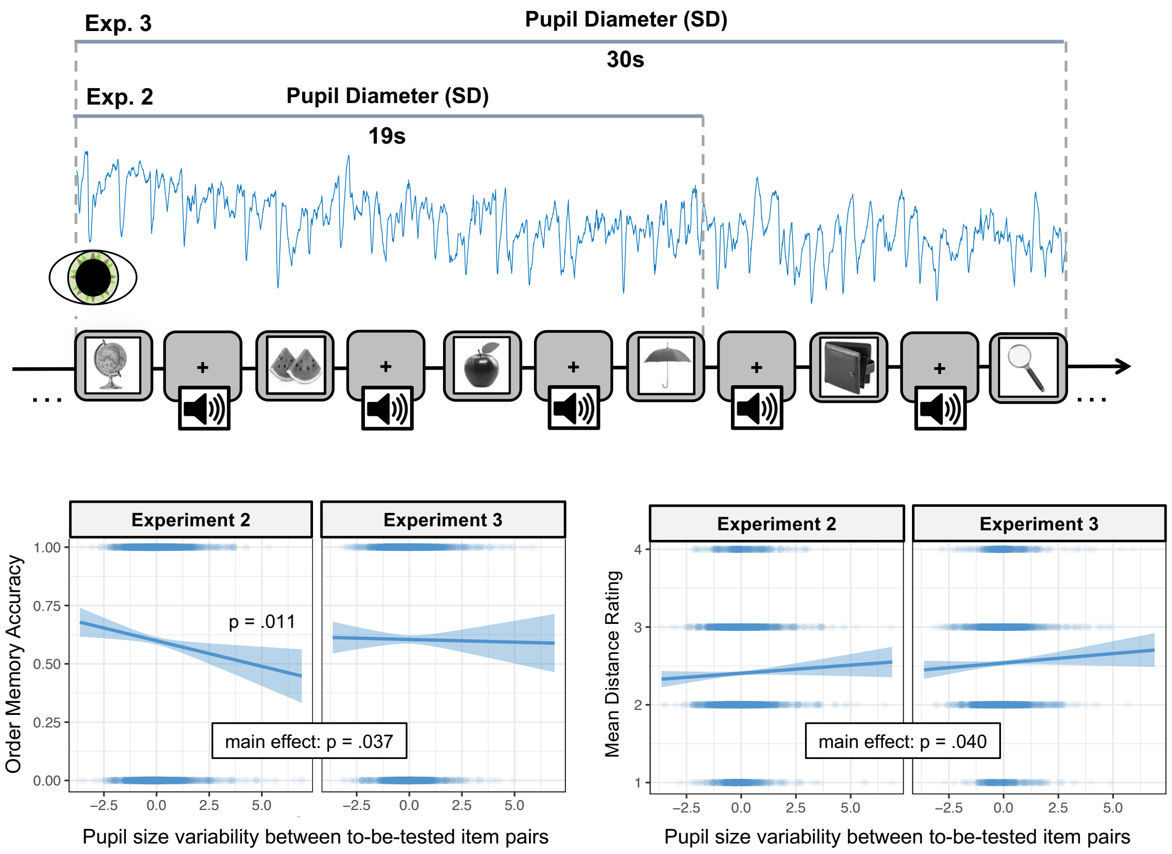

Supplementary Figure 5. The temporal stability of pupil-linked arousal states facilitates temporal memory integration. (Top Panel) To assess variability in pupil-linked arousal fluctuations across each sequence, we measured the standard deviation (SD) of pupil diameter values across all of the samples between to-be-tested item pairs. This time period was measured from the onset of the first image from a pair to the offset of the second image from that pair. In Experiment 2, the temporal distance between item pairs was 19 seconds, and in Experiment 3, the temporal distance between item pairs was 30 seconds. (Left Bottom Panel) Mixed effect linear modeling revealed a significant relationship between temporal recency discrimination and pupil size variability, such that participants were better at remembering the order of item pairs if there had been less variable changes in pupil size between those items pairs at encoding. This pupil-memory effect was significant when data from both eye-tracking studies were collapsed. (Right Bottom Panel) Linear regression models also revealed a significant relationship between temporal distance ratings and pupil size variability, such that participants were more likely to remember item pairs as having appeared closer together in time if there had been less variability in pupil diameter between those pairs at encoding. This pupil-memory effect was significant when data from both eye-tracking studies were collapsed. Shaded blue areas represent 95% confidence intervals. Overlaid dots represent individual participants (Experiment 2: $n = 35$; Experiment 3: $n = 30$). P-values are two-tailed and no adjustments for multiple comparisons were made. The pattern of results were similar across the two eye-tracking studies for the correlation between pupil variability and temporal distance ratings. Source data are provided as a Source Data file.

Supplementary References

1 Kaiser, H. F. The application of electronic computers to factor analysis. *Educational and psychological measurement* **20**, 141-151 (1960).

2 Aston-Jones, G. & Cohen, J. D. An integrative theory of locus coeruleus-norepinephrine function: Adaptive gain and optimal performance. *Annual Review of Neuroscience* **28**, 403-450 (2005).
