# Supplementary material for: Pupil-linked arousal signals track the temporal organization of events in memory
Source: Nat Commun. 2020 Aug 11;11:4007. doi: 10.1038/s41467-020-17851-9 (PMC7421896; doi:10.1038/s41467-020-17851-9)
Supplement: Supplementary file 3 — Reporting Summary [file 41467_2020_17851_MOESM3_ESM.pdf]

nature research

Corresponding author(s): Lila Davachi

Last updated by author(s): 2020/06/08

Reporting Summary

Nature Research wishes to improve the reproducibility of the work that we publish. This form provides structure for consistency and transparency in reporting. For further information on Nature Research policies, see Authors & Referees and the Editorial Policy Checklist.

Statistics

For all statistical analyses, confirm that the following items are present in the figure legend, table legend, main text, or Methods section.

n/a	Confirmed	
☐	☒	The exact sample size (*n*) for each experimental group/condition, given as a discrete number and unit of measurement
☐	☒	A statement on whether measurements were taken from distinct samples or whether the same sample was measured repeatedly
☐	☒	The statistical test(s) used AND whether they are one- or two-sided<br>*Only common tests should be described solely by name; describe more complex techniques in the Methods section.*
☐	☒	A description of all covariates tested
☐	☒	A description of any assumptions or corrections, such as tests of normality and adjustment for multiple comparisons
☐	☒	A full description of the statistical parameters including central tendency (e.g. means) or other basic estimates (e.g. regression coefficient) AND variation (e.g. standard deviation) or associated estimates of uncertainty (e.g. confidence intervals)
☐	☒	For null hypothesis testing, the test statistic (e.g. *F*, *t*, *r*) with confidence intervals, effect sizes, degrees of freedom and *P* value noted<br>*Give P values as exact values whenever suitable.*
☒	☐	For Bayesian analysis, information on the choice of priors and Markov chain Monte Carlo settings
☒	☐	For hierarchical and complex designs, identification of the appropriate level for tests and full reporting of outcomes
☐	☒	Estimates of effect sizes (e.g. Cohen's *d*, Pearson's *r*), indicating how they were calculated

Our web collection on statistics for biologists contains articles on many of the points above.

Software and code

Policy information about availability of computer code

Data collection	E.Prime 3.0; EyeLink 1000; G*power (sample size estimation) prior to data collection
Data analysis	SPSS Version 25; R; R Studio; Matlab R2019A

For manuscripts utilizing custom algorithms or software that are central to the research but not yet described in published literature, software must be made available to editors/reviewers. We strongly encourage code deposition in a community repository (e.g. GitHub). See the Nature Research guidelines for submitting code & software for further information.

Data

Policy information about availability of data

All manuscripts must include a data availability statement. This statement should provide the following information, where applicable:

- Accession codes, unique identifiers, or web links for publicly available datasets
- A list of figures that have associated raw data
- A description of any restrictions on data availability

All behavioral and eye-tracking data are publicly available on the Open Science Framework website '4QZNX [DOI 10.17605/OSF.IO/4QZNX]'. The source data underlying Figs 2-4, Fig. 5a-d, Fig. 6c/d, Fig. 7a/b, Supplementary Figs 2a/b, and Supplementary Figs 3-5 are provided as a Source Data file. A reporting summary for this article is available as a Supplementary Information file.

Field-specific reporting

Please select the one below that is the best fit for your research. If you are not sure, read the appropriate sections before making your selection.

☐ Life sciences ☒ Behavioural & social sciences ☐ Ecological, evolutionary & environmental sciences

For a reference copy of the document with all sections, see nature.com/documents/nr-reporting-summary-flat.pdf

Behavioural & social sciences study design

All studies must disclose on these points even when the disclosure is negative.

Study description

In this study, we performed three experiments to examine how an auditory context shift, or 'event boundary', within an item sequences influenced subsequent memory for the source information of those items (ear sounds were played in) as well as the objective temporal order of selected test item pairs and the subjective temporal distance between those same pairs. We also performed eye-tracking in two of those experiments so that we could measure fluctuations in autonomic arousal, indexed by pupil size/dilation, across sequence encoding. We then performed a PCA analysis to decompose the pupil dilation response to event boundaries into different temporal characteristics. To test our central hypotheses, we examined how 'loading' onto these different pupil components, or how much individuals exhibited those temporal patterns, at boundaries corresponded with boundary-related impairments in temporal order memory and more expanded retrospective estimates of temporal distance.

Research sample

Healthy young adults were were recruited from the New York University Psychology Subject Pool and nearby community to participate in this experiment. The study sample was representative of the area and can be broken down as follows. Experiment 1: Thirty-four individuals (23 women; Mage = 23.26, SDage = 4.52); Experiment 2: Thirty-five individuals (24 women; Mage = 22.57, SDage = 4.24); Experiment 3: Thirty individuals (21 women; Mage = 23.87, SDage = 5.55).

Sampling strategy

The sampling procedure was performed both randomly and for convenience (i.e., participants were not explicitly randomly assigned to one of the experiments according to a scheme). A power analysis was performed on data from a similar event boundary experiment to estimate the appropriate sample size (Dubrow and Davachi, 2013). With an alpha = .05 and power = .80, we needed 28 participants to obtain a large effect size (d = .80; Cohen's criteria) for the temporal order memory effect (G*Power 3.1). Additional participants were recruited in case of poor memory performance, potential withdrawal from experiment, or an inability to perform the task. We also expected overall temporal memory performance to be worse in the current experiment compared to the results reported in Dubrow and Davachi (2013), given that the sequence lists had eight additional items and there was a shorter lag between to-be-tested item pairs (two in this study vs. three items in Dubrow and Davachi, 2013)

Data collection

The single-session behavioral experiments took approximately 2 hours to complete. Each session started with acquiring informed consent. Participants then filled out a demographics question form (e.g., age, gender etc.). At the beginning of the task, participants were given instructions about the auditory event boundary experiment and familiarized with the eye-tracking procedure (including a 9-point eye position calibration). During the task, pupil data was measured continuously at 250 Hz during the sequence encoding segments of the task. A single experimenter remained in the room for the entire session so that he/she could monitor the eye recordings and ensure the participant didn't fall asleep. The experimenters were not blind to the hypotheses.

Timing

Data collection started in July of 2017 and ended in March of 2018.

Data exclusions

Experiment 1: For the source memory analysis (but not the two temporal memory tests), six participants were excluded from data analysis due to a programming error.

For the two eye-tracking studies, data loss of 50% for any given block was pre-determined as an exclusion criterion.

Experiment 2: Five participants were excluded from data analysis. Three participants withdrew mid-way through the experiment, one participant failed to follow task instructions, and the eye-tracker malfunctioned for one participant.

Experiment 3: A total of eight participants were excluded from data analysis. Four participants had poor eye-tracking quality (fewer than 50% valid samples) and four participants withdrew mid-way through the experiment.

Non-participation

As listed above, a total of 7 participants withdrew from the experiment after providing informed consent. Participants all stated that they were either too tired or bored to continue.

Randomization

The studies were conducted in succession, so data was collected at different timepoints. Thus, the groups are independent; post hoc comparisons were not conducted across experiments, therefore time is not a confound. The average age of participants was virtually the same across studies (Exp 1: mean = 23; Exp 2: mean: 23; Exp 3: mean = 24) and the proportion of women was also nearly identical (Exp 1: 69% of sample; Exp 2: mean: 70% of sample; Exp 3: 68% of sample). Additionally, age and sex were not covariates of interest. Nevertheless, these variables were well matched across studies.

Reporting for specific materials, systems and methods

We require information from authors about some types of materials, experimental systems and methods used in many studies. Here, indicate whether each material, system or method listed is relevant to your study. If you are not sure if a list item applies to your research, read the appropriate section before selecting a response.

Materials & experimental systems

n/a	Involved in the study
☒	☐ Antibodies
☒	☐ Eukaryotic cell lines
☒	☐ Palaeontology
☒	☐ Animals and other organisms
☐	☒ Human research participants
☒	☐ Clinical data

Methods

n/a	Involved in the study
☒	☐ ChIP-seq
☒	☐ Flow cytometry
☒	☐ MRI-based neuroimaging

Human research participants

Policy information about studies involving human research participants

Population characteristics	See above
Recruitment	Participants were recruited from NYU and the nearby community via IRB-approved flyers posted in the NYU Psychology building. Participants were also recruited using the NYU Psychology Subject Pool website, which is implemented on SONA Systems.
Ethics oversight	The study protocol was approved by the New York University Institutional Review Board

Note that full information on the approval of the study protocol must also be provided in the manuscript.
